# Expression of influenza A virus-derived peptides on a rotavirus VP6-based delivery platform

**DOI:** 10.1007/s00705-020-04847-5

**Published:** 2020-10-16

**Authors:** Stina Gröhn, Suvi Heinimäki, Kirsi Tamminen, Vesna Blazevic

**Affiliations:** Faculty of Medicine and Health Technology, Vaccine Development and Immunology/Vaccine Research Center, Tampere University, Arvo Ylpön katu 34, FI-33520 Tampere, Finland

## Abstract

Recombinant protein technology enables the engineering of modern vaccines composed of a carrier protein displaying poorly immunogenic heterologous antigens. One promising carrier is based on the rotavirus inner-capsid VP6 protein. We explored different VP6 insertion sites for the presentation of two peptides (23 and 140 amino acids) derived from the M2 and HA genes of influenza A virus. Both termini and three surface loops of VP6 were successfully exploited as genetic fusion sites, as demonstrated by the expression of the fusion proteins. However, further studies are needed to assess the morphology and immunogenicity of these constructs.

Modern bioengineering technologies have facilitated the design of subunit vaccines as safe and affordable alternatives to conventional vaccines [1]. At the frontline of the new alternatives are self-assembling protein-derived nanoparticles, which can also be employed as platforms or nanocarriers. These particles can be decorated with heterologous antigens, including peptides, protein domains, or (poly)saccharides with significant biological relevance but poor immunogenicity on their own due to small size, incorrect configuration, or lack of stability [1]. A protein functioning as a carrier typically has a highly ordered particulate structure and a size that is optimal for uptake by antigen-presenting cells [2], efficiently inducing immune responses against the heteroantigens presented on the platform. One of the most frequently used platform technologies is based on virus-like particles [3] that have been successfully decorated with antigens of various origins, such as hepatitis B virus [4] and influenza virus [5]. Another promising nanocarrier is based on the rotavirus (RV) medium-layer VP6 protein, which forms various nanostructures when exposed to different physiological conditions [6]. These nanostructures are extremely immunogenic and have been shown to possess adjuvant properties when co-administered with antigens *in vitro* and *in vivo* [7–10].

It has been demonstrated previously that inserting foreign sequences by genetic fusion to the surface loops or N-terminus (N-t) of VP6 does not affect the structure of the VP6 monomer, allowing the insertion of at least a 15-amino-acid (aa) peptide [11, 12]. In the present study, we aimed to evaluate the effect of insertions of different sizes on the expression of VP6 fusion proteins (FPs) by cloning these heterologous sequences at different sites of VP6. To test the capacity of VP6 to accommodate foreign antigenic sequences, we selected the extracellular domain of the M2 protein (M2e) and a stem fragment of hemagglutinin (HA) derived from influenza A virus as model antigens. These antigens are promising universal vaccine candidates due to their high degree of conservation [13, 14]. The HA stem fragment is known to induce broadly protective neutralizing antibodies [14–16], whereas M2e induces cross-protective antibodies and CD8^+^ T cells [13, 17]. This study shows that VP6 can carry antigens of different sizes at several insertion sites. However, the insertion site affects the FP expression level and secretion pattern.

The internal positions (aa 171, aa 301 and aa 311 located in surface loops) in the 397-aa sequence of VP6 (RVA/Hu-wt/RUS/Novosibirsk/Nov09-D83/2009/G1P[8]) were selected based on a SWISS-MODEL analysis of the spatial conformation of the VP6 structure deposited by Mathieu et al. [18] in the Protein Data Bank (PDB code: 1qhd ) (Fig.1A). Seven recombinant FPs (FP1-FP7) were designed by inserting the M2e peptide and/or HA stem fragment polypeptide to the surface loops, C-terminus (C-t), or N-t of VP6 by genetic fusion (Fig. 1B). A 23-aa M2e peptide of human origin based on the consensus sequence SLLTEVETPIRNEWGCRCNDSSD [13] was fused to the VP6 sequence at aa 171 (FP1, 48 kDa) and N-t (FP2, 48 kDa). Additionally, FPs containing three copies of the M2e sequence, of human, swine (SLLTEVETPTRSEWECRCSDSSD, A/California/07/2009) and avian (type I consensus sequence SLLTEVETPTRNEWESRSSDSSD [19–21]) origin, either as a tandem repeat at aa 171 (FP3, 56 kDa), or as individual peptides at aa 171, aa 301 and aa 311 (FP4, 56 kDa) were created. An HA stem fragment polypeptide (140 aa) was inserted at N-t (FP5, 62 kDa) and C-t (FP6, 62 kDa) of VP6. The HA stem fragment consists of aa 18-41 and aa 290-323 of subunit HA1 and aa 41-113 of subunit HA2 from the influenza A virus H1N1 HA (A/Puerto Rico/8/34 subtype) connected by GSA and GSAGSA linkers, and it mimics the structure of the epitope of the HA stem, inducing broadly neutralizing antibodies [14]. In addition, FP7 (65 kDa) was designed to contain both the human M2e peptide at aa 171 and the HA stem fragment polypeptide at N-t. Each insert was fused to the VP6 backbone with common flexible linkers (G_4_S)_1-3_ to improve the display of the insert and to sustain the correct folding of the carrier protein. The DNAs coding for the FPs were synthetically subcloned into pFastBac™ 1 baculoviral transfer vectors by GeneART Gene Synthesis service (Germany).

**Fig. 1:**
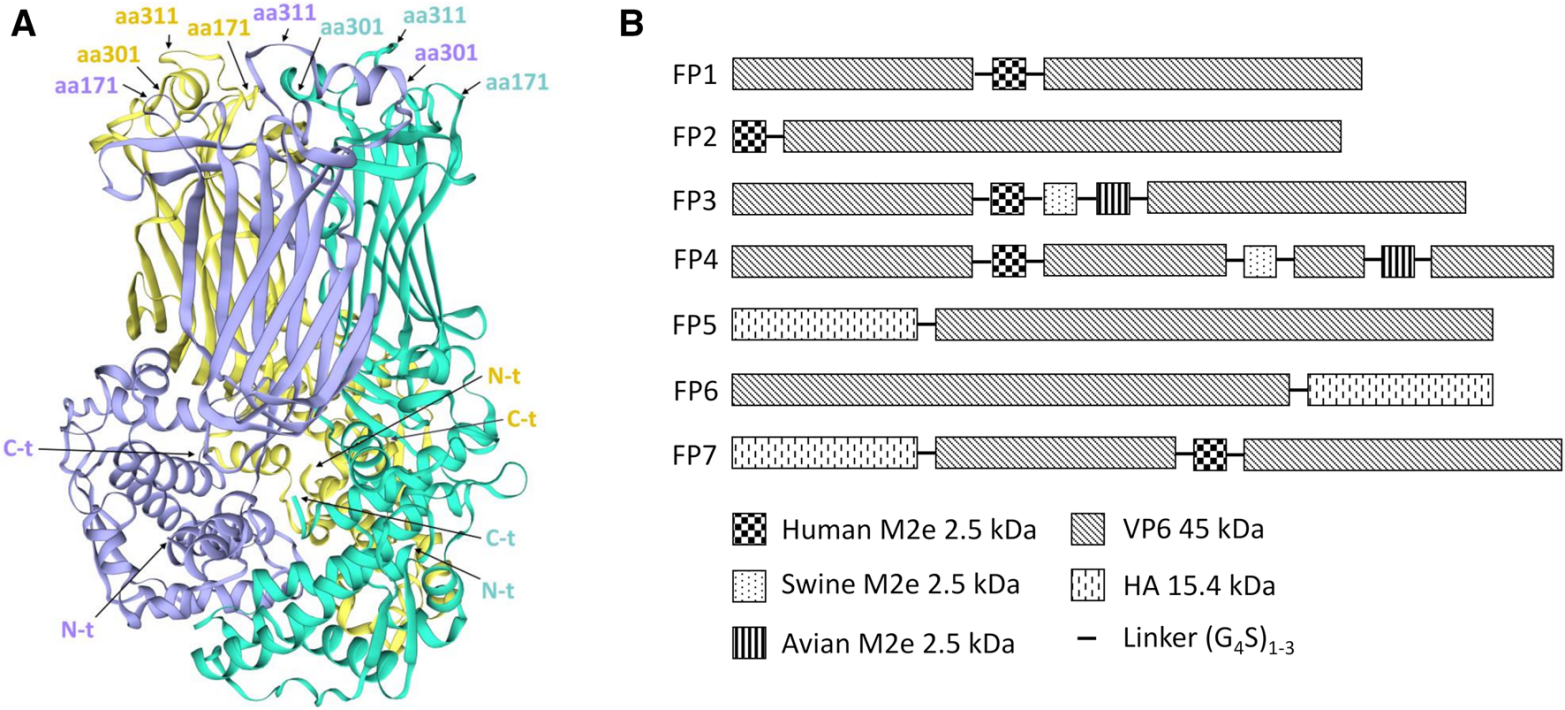
Schematic representation of insertion sites on the rotavirus VP6 protein and fusion protein design. (A) VP6 homotrimer with different insertions sites at aa 171, aa 301 and aa 311 located in different surface loops, N-terminus (N-t), and C-terminus (C-t), which are indicated by arrows. The VP6 structure was modelled in SWISS-MODEL using the crystal structure solved by Mathieu et al. [18] as a template (PDB code: 1qhd). (B) Schematic representation of seven fusion proteins (FPs) carrying the M2e peptide and/or the HA stem fragment polypeptide from influenza A virus at the N- or C-terminus or internal sites of VP6. Flexible linkers were used to connect independent fragments.

Recombinant baculovirus (rBV) stocks were generated as described before [22]. Briefly, recombinant bacmids were constructed using the Bac-to-Bac™ Baculovirus Expression System (Gibco, Carlsbad, USA). *Spodoptera frugiperda* (Sf9) insect cells were transfected with bacmid DNA using Cellfectin II transfection reagent (Gibco), and after 4 days, the P0 rBV stocks were collected. The P0 stocks were used to infect Sf9 cells (2 × 10^6^ cells/ml) to generate P1 stocks. The titers of the rBV stocks were determined using a BacPAK™ Baculovirus Rapid Titer Kit (TaKaRa, Mountain View, USA).

The optimal conditions for expression of each FP were determined by comparing different insect cell lines, Sf9 and High Five™ (*Trichoplusia ni*), cell densities (1 × 10^6^ and 2 × 10^6^ cells/ml), multiplicity of infection (MOI) values (1, 5 and 10 infectious units (IFU)/cell), and cultivation times (3-7 dpi). Sf9 cells were cultured in Sf-900™ III SFM medium (Gibco), and High Five™ cells in Insect-XPRESS™ Protein-free Insect Cell Medium (Lonza, Walkersville, USA). The optimization was carried out in 6-well plates (Nunc A/S, Roskilde, Denmark) with a volume of 2 ml and in culture flasks (Corning Inc., Corning, USA) with a volume of 30ml at +27°C in an orbital shaker (122 rpm). The optimal time for culture harvesting was deduced by monitoring the cytopathic effect using trypan blue staining (Countess™, Invitrogen, Carlsbad, USA). The cells were separated from the supernatant by centrifugation (1000 × *g* at +4°C for 20 min), and the pellets and supernatants were stored at -20° C and +4°C, respectively, for further characterization.

Protein expression from the pellet and supernatant of each culture was analyzed by sodium dodecyl sulfate polyacrylamide gel electrophoresis (SDS-PAGE) using Mini Protean TGX Precast gels (Bio-Rad Laboratories, Hercules, USA) and PageBlue Protein Staining Solution (Thermo Scientific, Rockford, USA). To verify the presence of FPs, the supernatants and pellets were analyzed by VP6-specific Western blotting. The proteins separated by SDS-PAGE were transferred onto a nitrocellulose membrane (Bio-Rad) using a Trans-Blot® SD Semi-Dry Transfer Cell (Bio-Rad). The membranes were immunoblotted in an iBind Flex Western Device (Invitrogen) using 1:500-diluted mouse RV VP6 antibody IgG2a kappa (Novus Biologicals, Centennial, USA) and 1:1000-diluted goat anti-mouse IgG-HRP (Sigma-Aldrich, St. Louis, USA). The immunoblots were developed using an Opti-4CN Substrate Kit (Bio-Rad). The FPs were also subjected to insert-specific Western blotting using anti-M2 (1:2000-diluted mouse influenza A M2 monoclonal antibody (14C2), Invitrogen) or anti-HA (1:500-diluted rabbit influenza A H1N1 hemagglutinin antibody, Sino Biological, Wayne, USA) antibodies and 1:1000-diluted goat anti-mouse (Sigma-Aldrich) or 1:5000-diluted goat anti-rabbit (Abcam, Cambridge, UK) IgG-HRP, respectively. In addition, the ability of the FPs to trimerize was examined by SDS-PAGE under non-reducing conditions as described by Peralta et al. [12], followed by VP6-specific Western blotting.

The expression of FPs was analyzed by SDS-PAGE for each culturing condition described above. An MOI value of 1 and cell density of 1.0 × 10^6^ cells/ml were determined to be optimal for all FPs, as no significant improvements in protein yields were observed when using higher MOI values or cell density at the time of infection (data not shown). A cultivation time of 6 days was determined to be optimal for all FPs based on expression levels and the number of dead cells in the cultures (>80%). The optimal cell line was deduced by observing the localization of the FPs (Fig. 2A). All FPs with an internal insert (FP1, FP3, FP4, FP7) were predominantly intracellularly located with a minor quantity of protein secreted. In comparison, the FPs with an insert at either terminus (FP2, FP5 and FP6) were secreted in higher quantities into the cell culture supernatant. Therefore, the Sf9 cell line was determined to be optimal for intracellularly located FPs, while the High Five™ cell line was ideal for secreted FPs. The optimal production conditions determined for all FPs are presented in Table 1.

**Fig. 2:**
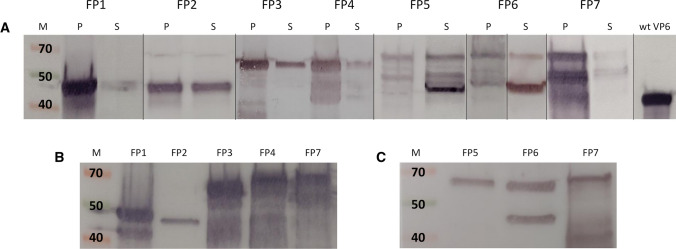
Expression of VP6 fusion proteins carrying influenza A virus-derived antigens. (A) Pellet (P) and supernatant (S) samples from fusion proteins FP1-FP7 immunoblotted with VP6-specific antibody. M (molecular weight)-marker in kilodaltons (kDa); wtVP6, purified "wild-type" VP6 protein (45 kDa) used as a positive immunoblotting control. The figure is composed of blots from several different experiments, which are separated by lines. All samples, however, were subjected to same procedures during the experiments. (B) Pellet samples of FP1, FP3, FP4 and FP7 and the supernatant sample of FP2 immunoblotted with M2-specific antibody. (C) The supernatant samples of FP5 and FP6 and the pellet sample of FP7 immunoblotted with HA-specific antibody

**Table 1:** Optimal production conditions for all fusion proteins (FPs)

Fusion protein	Cell line	Cell density at the time of infection (cells/ml)	rBV stock titer (IFU/ml)	MOI	Culture time (dpi)	Predominant localization of the fusion protein
FP1	Sf9	1.0 × 10^6^	3.33 × 10^8^	1	6	P
FP2	High Five™	1.0 × 10^6^	8.27 × 10^7^	1	6	P/S
FP3	Sf9	1.0 × 10^6^	2.13 × 10^8^	1	6	P
FP4	Sf9	1.0 × 10^6^	5.47 × 10^7^	1	6	P
FP5	High Five™	1.0 × 10^6^	5.07 × 10^7^	1	6	P/S
FP6	High Five™	1.0 × 10^6^	4.80 × 10^7^	1	6	P/S
FP7	Sf9	1.0 × 10^6^	1.60 × 10^8^	1	6	P

MOI, multiplicity of infection; P, pellet; S, supernatant

The supernatants and pellets were further analyzed by immunoblotting to confirm the expression of each FP and to determine its size and antigenicity. All FPs exhibited a band of the expected size, when detected using a VP6-specific antibody, demonstrating successful expression of all products (Fig. 2A). A pronounced cleavage in FP5, FP6 and FP7 resulted in additional smaller proteins (~50 kDa), suggesting that a large terminal insert might expose these FPs to partial proteolysis. FPs containing M2e were also detected with an M2-specific antibody (Fig. 2B), and FPs containing the HA stem fragment with an HA-specific antibody (Fig. 2C), further confirming the presence of the correct insertions. According to the preliminary trimerization analysis, FP1, FP2, FP3, FP5 and FP6 were capable of trimerization (data not shown).

We have previously shown not only that VP6 is a potential candidate for a non-live RV vaccine [22, 23] but that it also possesses favorable adjuvant properties promoting the uptake and immunogenicity of the co-administered antigens [7–10]. Due to these immunomodulatory functions, VP6 could serve as a potential carrier for foreign antigens with poor immunogenicity. The objectives of this study were to employ VP6 as a carrier for the influenza A virus M2e and HA stem fragment and to investigate the most appropriate insertion sites and conditions for successful expression of the FPs.

We found an MOI value of 1, cell density of 1.0 × 10^6^ cells/ml and cultivation time of 6 days to be ideal for all FPs. These observations are in accordance with previous studies [24], suggesting that a low MOI should be used with a low cell density, as only a fraction of the cell population is initially infected, and thus, a longer cultivation time is needed to support viral progeny and recombinant protein production, respectively. Sf9 cells were determined to be optimal for non-secreted FP production based on a higher expression level of FPs in the cell pellet, as observed previously [25]. High Five™ cells, in turn, were superior for secreted FPs, as the expression levels were generally higher in High Five™ supernatants than in Sf9 supernatants (data not shown). In support of this, it has been shown previously that High Five™ cells are more suitable for the expression of secreted proteins [25].

According to the immunoblotting results, FPs with inserts at internal positions were predominantly intracellularly located, and FPs with terminal insertions were primarily secreted but also found in the pellets, similarly to wild-type VP6 [22, 23]. The intracellular accumulation of FPs may be due to internal insertions causing potential steric hindrance in the VP6 structure, thereby hampering the correct folding, trimerization, and/or oligomerization of VP6 [26]. The oligomerization state of each FP warrants further studies, but according to our preliminary results, inserts at aa 171, C-t or N-t do not prevent trimerization. Concurringly, Peralta et al. showed that an insert at the N-t does not affect the ability of VP6s to trimerize [12]. However, they could not detect trimers formed by VP6 carrying an insert at aa 171, aa 301 or aa 311. Despite the fact that some insertions might inhibit the trimerization of VP6 [12, 18], we have recently shown that a lack of a high-order structures does not negatively affect VP6 uptake and presentation by murine bone-marrow-derived dendritic cells *in vitro* [27].

The modifiability of protein-based vaccine platforms enables the decoration of carrier proteins with a large range of antigens, thus offering virtually unlimited vaccine opportunities. Additionally, platform technology ideally streamlines the manufacturing process, leading to potentially lower capital and operating costs. Since platform technology allows rapid and simple modifications with selected antigens, it could facilitate development of new vaccines against rapidly spreading viruses, such as influenza virus and coronaviruses. The present study demonstrates that VP6 has great potential to function as a platform for heterologous antigen display and delivery, as VP6 was capable of carrying 23- and 140-aa sequences at several insertion sites. In the light of recent findings, all FPs present the inserted antigens equally well. Therefore, we cannot make final statements on the best insertion sites, as the effect of insertions on VP6 structure and epitope accessibility has not yet been thoroughly investigated. Further studies are needed to determine the morphology and *in vivo* immunogenicity of the FPs, and thus the optimal insertion sites in VP6.
